# Enhancing Well-Being: A Comparative Study of Virtual Reality Chromotherapy Rooms with Static, Dynamic, and Empty Environments

**DOI:** 10.3390/s24061732

**Published:** 2024-03-07

**Authors:** Mine Dastan, Marina Ricci, Fabio Vangi, Michele Fiorentino

**Affiliations:** Department of Mechanics, Mathematics, and Management, Polytechnic University of Bari, 70126 Bari, Italy; marina.ricci@poliba.it (M.R.); f.vangi@phd.poliba.it (F.V.)

**Keywords:** color therapy, immersive virtual worlds, stress management, wellbeing, user presence, interaction design

## Abstract

Chromotherapy rooms (CRs) are physical spaces with colored lights able to enhance an individual’s mood, well-being, and, in the long term, their health. Virtual reality technology can be used to implement CR (VRCRs) and provide higher flexibility at lower costs. However, existing VRCRs are limited to a few use cases, and they do not fully explore the potential and pitfalls of the technology. This work contributes by comparing three VRCR designs: empty, static, and dynamic. Empty is just a void but a blue-colored environment. Static adds static abstract graphics (flowers and sea texture), and dynamic adds dynamic elements (animated star particle systems, fractals, and ocean flow). All conditions include relaxing low-beta and ocean sounds. We conducted a between-subject experiment (*n* = 30) with the three conditions. Subjects compiled a self-perceived questionnaire and a mathematical stress test before and after the VRCR experience. The results demonstrated that the dynamic condition provided a higher sense of presence, while the self-perceived stress level was insignificant. Dynamic VR conditions are perceived as having a shorter duration, and participants declared that they felt more involved and engaged than in the other conditions. Overall, the study demonstrated that VRCRs have a non-trivial behavior and need further study of their design, especially considering their role in a future where VR will be an everyday working interface.

## 1. Introduction

Chromotherapy is a treatment that exploits the chromatic properties of visible light to treat diseases such as aches, nosebleeds, and burns [1]. The color was often used for patient diagnostics for centuries; then, appropriate colored rooms—defined as ‘chromotherapy rooms’—were used for healing [2]. Chromotherapy rooms (CRs), also known as color therapy rooms, are places to enhance an individual’s mood, well-being, and health with the use of colored lights [3]. The rationale is that each color has a unique energy that affects the person’s emotional and physical condition [4].

Although experimental studies showed that CR influences cognitive tasks and performance, time perception, emotions, memory, and pain perception [5], color therapy is still not widely investigated in medicine. In addition to softly lit atmospheres with walls, floors, and ceilings made of color-emitted materials, CRs are also commonly enhanced with other sensory stimuli, such as calming background music [6].

Despite their effects, physical CRs are expensive to build and maintain and have reduced capacity.

The use of virtual reality (VR) technology, which makes use of interactive computer-generated immersive environments, can provide customers with CR experiences (VRCR), as it has been shown to be effective for anxiety and phobia treatments [7,8]. Previous research also evidenced that VR provides easy-to-carry affordable options to physical CRs for anxiety management anywhere possible, like workplaces and homes. VRCRs will play an important role in the short-term future, where VR will be a common and everyday working interface.

In previous studies, it has been shown that dynamic content within a virtual environment may have a positive impact over static content [9,10,11]. However, the grounds and the guidelines related to these VRCR design elements are still unknown. Our research aims to provide valuable insights for developing effective and tailored VRCR interventions by identifying contradictory results and discussing future applications.

Therefore, in this study, we compare the use of empty, static, and animated VR CRs and their impact on user presence and self-perceived stress. The contribution of this paper is threefold: (1) implement and evaluate a novel VRCR design; (2) compare the impact of different approaches (static, dynamic, and empty conditions) on user presence and self-perceived stress; and (3) draw lessons learned for future works related to VR CRs.

## 2. Related Works

Chromotherapy, an alternative therapeutic modality rooted in the principles of color psychology, has garnered attention for its potential role in stress relief and overall mental well-being. Research in the field suggests that exposure to certain colors can have significant physiological and psychological effects on individuals. For instance, a study by Labrecque and Milne [12] found that blue has calming effects, leading to reductions in heart rate and blood pressure, thereby alleviating stress. Similarly, the work of Alvarsson et al. [13] demonstrated that green environments, associated with nature, can positively influence mood and stress recovery. The physiological impact of colors is further supported by a study conducted by Kwallek et al. [14] which indicated that warm colors like red may stimulate arousal and energy. By integrating insights from such scientific investigations, chromotherapy emerges as a promising avenue for stress relief, providing a non-invasive and complementary approach to conventional stress management strategies. As our understanding of the physiological and psychological impacts of color continues to advance, the potential applications of chromotherapy in stress reduction warrant further exploration and consideration in holistic well-being practices.

We divide the state-of-the-art into two sections: the first analyzes physical and virtual CR in the literature, and the second focuses on the design elements for VRCR.

### 2.1. Physical and Virtual Chromotherapy Rooms

Blasco M. et al. [7] pioneered research on VRCRs comparing physical and virtual CRs and assessing the stress levels of two groups. The VRCR was a replica of a real physical room (6 m^2^) with the same place to sit. The experiment was between subject participants (two groups). The results demonstrated that there are no significant differences in the EEG parameters of the participants (*n* = 20) between the VR and the traditional room, indicating that the VR version can be a low-cost alternative to CRs. They later presented 360 videos of the chromotherapy experience [8] where the participants were immersed in the natural ambiance and sounds.

Minguillon J. et al. [3] conducted a study in which 12 healthy volunteers were stressed and then performed a relaxation session in a CR with blue (test group) or white (control group) lighting. Their study demonstrated that blue light accelerates the relaxation process. Colors such as blue can also be used to treat conditions such as sleep disorders, jet lag, seasonal affective disorder, and premenstrual syndrome [15].

Sembian N. et al. [16] explored the effectiveness of magenta in CRs. Magenta stimulates adrenaline and heart activity, the energy needed for power and self-realization, and strong but controlled passions. Paragas E. et al. [17] experimented with three group conditions, namely, red, green, and white lights, with 45 subjects in each group. Based on the results of the study, the green light improved older adults’ orientation, recall, attention, and calculation scores.

Overall, the existing approaches to VRCRs are limited in number and are commonly designed as pseudo-static rooms, replicas of the physical CRs, or naturalistic 360 videos with low levels of dynamism, interactivity, and presence. Current VRCR knowledge is limited, especially as the existing approaches do not explore, at best, the possibility of VRCRs providing dynamic experiences.

### 2.2. Dynamic and Blue Scenery for Relaxation

Veiling W. et al. [18] studied dynamic animated elements such as air bubbles, circles, and harmonious melodies and conducted a user experiment on 50 patients with anxiety, psychotic, depressive, and bipolar disorders. The results demonstrated that relaxation in VR with immersive 360° nature videos resulted in a significantly greater reduction in total negative affective state than the standard relaxation method, which is 360 videos.

Gonzales D. et al. [19] presented interactive stress reduction sessions between 50 participants in two groups: a real CR experience and a replica of CR in VR. The results demonstrated that augmenting virtual worlds with elements simultaneously experienced in the real world may increase the sense of presence and enhance simulation efficacy, e.g., increased stress reduction, when compared to the use of VR only.

Soyka et al. [20] developed an underwater environment with animated elements (jellyfish) and a combination of breathing techniques to help participants (*n* = 21) relax in the virtual environment. The results demonstrated that their virtual underwater simulation was rated as more fun and more likely to be used at home than a traditional breathing technique. As a result, there is a wide body of scientific literature supporting how dynamic scenes with animated objects can have a positive impact on stress reduction and management.

Another aspect we consider for our design is the use of the color blue to encourage a sense of calmness and relaxation [3,21]. Kwallek N. et al. [22] conducted research demonstrating that the color blue promotes stress reduction and relaxation by stimulating the production of calming chemicals like serotonin in the human body. Blue is often perceived as a calming color that can have a psychological impact on individuals and contribute to relaxation [23].

Possible explanations include the blue color’s association with water and air environments such as the ocean and sky. Previous studies demonstrated that the influence of water has proved to be an important trigger for humans, whether in natural or VR scenes [24,25,26].

### 2.3. Impact of Simulated Natural Environments

Undoubtedly, exposure to natural environments positively influences stress recovery. Kjellgren A. et al. [27] studied the comparison of the real and simulated natural environments by performing a study where they tested the restorative effects of 30 min of relaxation in a natural environment with an indoor simulation of the same natural environment. A repeated-measure design was carried out, and 18 participants suffering from stress and/or burnout syndrome were counterbalanced into the two conditions. The results suggest that both environments facilitate stress reduction, with the natural environment additionally bringing increased energy, thus enhancing and promoting restoration.

While real natural environments effectively promote recovery from stress, virtual exposure to nature also positively affects stress [28,29]. For instance, Abdullah S. et al. [28] suggested that VR provides benefits for assessing and reducing stress levels. Thus, VR could be an effective technique for promoting relaxation, as they saw during the COVID-19 pandemic, when stress levels rose globally.

Wang et al. [29] instead explored the effects of different types of forest environments on forest therapy. Seven representative forest resting environments found in field research in Beijing were used as independent variables and were shown to subjects through a VR video. A between-subjects design was used in the experiment with 96 subjects, discovering that all seven different types of forest resting environments can produce stress relief effects to some extent. Different types of forest resting environments have different effects on relieving stress. Interestingly, the most natural environment does not have the most significant effect on stress relief, while a water landscape has a positive effect on stress relief.

## 3. The Design of the Experiment

**Figure 1:**
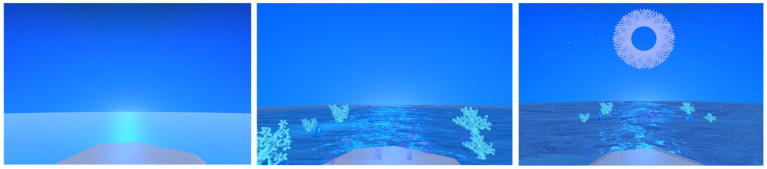
The three conditions: empty (**left**), static (**center**), and dynamic animating the static with star particle systems, fractals, and blue ocean flow (**right**) (Supplementary Materials Video S1).

The primary objective of our experiment is to investigate the impact of dynamic/static elements in VRCRs on self-perceived stress reduction and user preferences. Our main research question is “How do different scene designs in VRCR impact self-perceived stress and user presence?”. To address this inquiry and highlight the potential for developing more effective and immersive VRCR experiences, we conducted a comparative analysis of three conditions/scenes (1) empty (only color), (2) dynamic, and (3) static, as depicted in Figure 1.

Thus, we formulated three hypotheses:

- The dynamic VRCR increases user presence (H1) vs. static and empty.
- The dynamic VRCR reduces their self-perceived stress (H2) vs. static and empty.
- Users will feel immersed for a shorter time within the dynamic VRCR vs. static and empty (H3).

### 3.1. Implementation

We designed and implemented the empty/static/dynamic conditions as virtual scenes using Unity 3D and developed a standalone application on an Oculus Quest2 device (Meta Platforms, Inc., Menlo Park, CA, USA).

**Figure 2:**
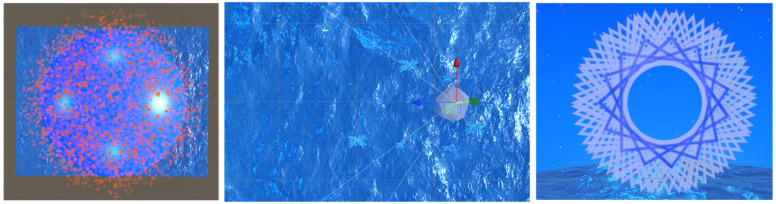
VRCR dynamic scene: top view of the Unity 3D Skydome; the red points are particle systems (**left**), top view of the ocean waves that move in the simulation and participant placement indicated with arrows (**center**), rotating fractal displayed in the frontal view of the participant (**right**).

The empty condition is a blue-colored environment with a sky and flat-colored sea with no additional elements; the static condition has a static-textured ocean with blue-colored sea corals and plants (see Figure 2). The dynamic condition adds animated sea waves, circles, fractals, and corals created with Unity shaders and particle systems, as shown in Figure 2, and includes relaxing low-beta and ocean sounds.

### 3.2. Participants

We conducted a between-subjects user experiment involving 30 unpaid healthy voluntary participants (63% male, 37% female) recruited from the university platforms of the Industrial Design and Mechanical Engineering Departments at the Polytechnic University of Bari, Italy. We first asked participants whether they had organic or psychopathological vulnerabilities. None of them presented any of these problems, and we included them in the study. The average age of the participants was 26, with a standard deviation of 3.37, ranging from 21 to 34 years. All the participants had previous experience with VR gained through university courses, with a VR familiarity level rating of 4.86 on a 1–7 Likert scale (SD = 1.55). However, none of them had ever experienced a virtual environment featuring relaxing, mindful, and color therapy elements. All participants provided informed consent before starting the experiment. The user test adhered to the ethical guidelines outlined in the American Psychological Association Code of Ethics.

### 3.3. Procedure and Measures

**Figure 3:**
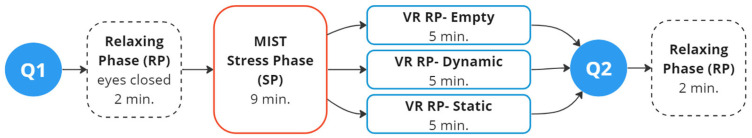
The phases of the experiment: (P1) Q1 self-perceived stress questionnaire, (P2) relaxing, (P3) MIST stress, (P4) VRCR divided into groups (dynamic/static/empty), (P5) Q2 self-perceived stress questionnaire, and (P6) the end of the experiment.

The subjects were randomly divided into three test groups (empty, dynamic, and static conditions) and followed the phases specified in Figure 3.

Each subject first receives the preliminary introductions and provides consent for participation. In the first phase, participants compile a Q1 self-perceived stress questionnaire [20,30]. In phase two, participants sit on a chair, close their eyes, and rest for 2 min in the relaxing phase.

**Figure 4:**
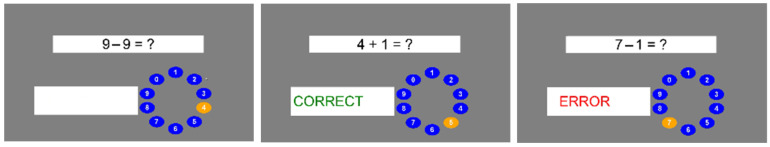
The Montreal Imaging Stress Test used in the test: The subject has 9 min to answer.

Subsequently, participants are stressed for 9 min by answering the Montreal Imaging Stress Test (MIST [31] multiple mathematical problems (see Figure 4), as used from multiple studies in the literature [7,8,32].

**Figure 5:**
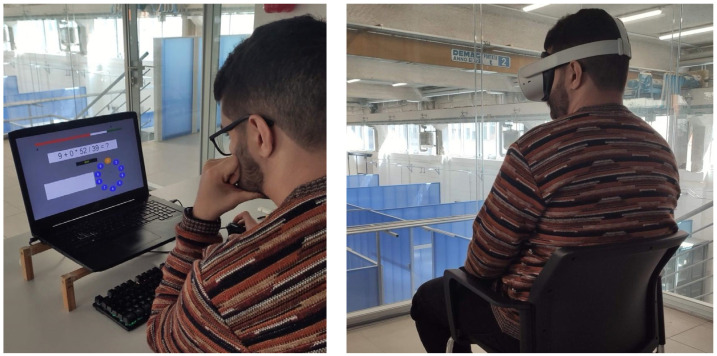
The flow of the user experiment: MIST stress phase and VR condition.

After the MIST test, participants wear the Oculus Quest 2 VR headset and experience their condition scenery for 5 min (see Figure 5). The participants are not aware of the duration of the VR environment and are left alone in a quiet place and monitored by us from outside of the room using a “shadowing technique”. Participants were not aware of the experiment’s objective and were not conditioned by the experimenters during the task.

**Figure 6:**
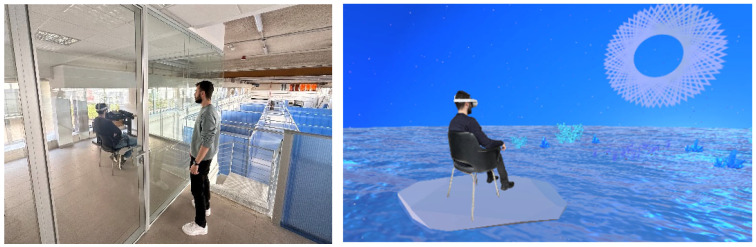
The observer supervises the experiment from outside of the room while the subject tries the VRCR (**left**), a simulated image of a subject immersed in the dynamic chromotherapy condition (**right**).

When the VR experience finishes, the participants are asked to remove the headset and compile the second Q2 self-perceived stress questionnaire and presence questionnaire (PQ) [33]. The test terminates with a 2 min relaxing phase to assess cybersickness and to adjust simulation effects. The experiment is continuously supervised by an observer who takes notes about the experiment’s events, as shown in Figure 6.

## 4. Results

The user experiment lasted 20 min, and we acquired 30 valid entries, without any unexpected events or interruptions. We divided the results for the self-perceived stress and the presence questionnaire results.

### 4.1. Self-Perceived Stress

We analyzed the data using three independent samples of the two-way repeated ANOVA-Friedmann test using the statistical tool JASP for each of the three conditions (empty, static, and dynamic).

**Figure 7:**
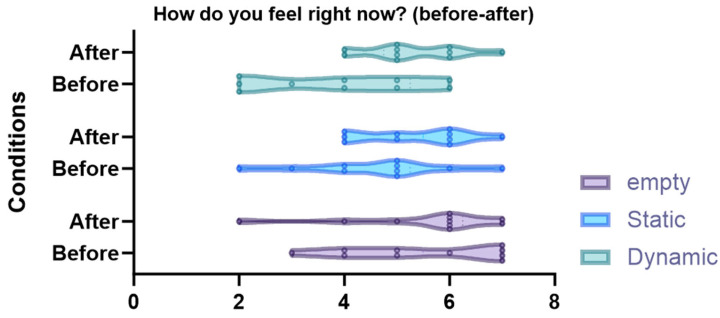
Violin plots and post-hoc comparisons of the before-test and after-VR self-perceived stress rates on a Likert scale from 1–7.

The self-perceived stress rate of the participants was not significant (*p* > 0.05), both before the test (*p* = 0.078) and after the VR (*p* = 0.911) (see Figure 7).

We also formulated other questions (on a 7-point Likert scale), as follows:

1. “Did you find the VR experience relaxing?” (*p* = 0.435)
2. “Was the experience fun or boring?” (*p* = 0.204)
3. “Would you do this experience at home for relaxing?” (*p* = 0.082)
4. “How difficult was it to relax with the presented elements in the scene?” (*p* = 0.227)
5. “How long was your VR experience?” (*p* = 0.465)

### 4.2. Presence

The presence questionnaire is composed of 10 questions on a Likert scale of 1–7.

We first applied the Kruskal–Wallis test to each question and then applied post-hoc comparison to analyze the relationship between groups, correcting for multiple comparisons using the Bonferroni correction. We used the violin plots to visualize the distribution of the data.

**Figure 8:**
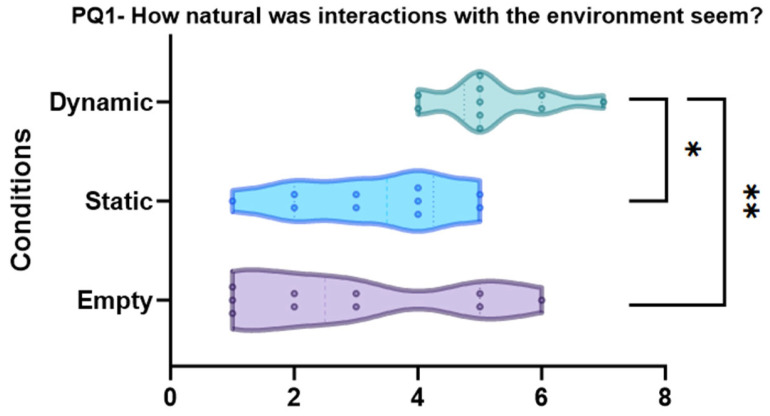
Violin plots and post-hoc comparisons for the presence question “How natural was interaction with the environment?”. Dynamic scene is significantly better than empty scene (pbonf = 0.004 **) and static conditions (pbonf = 0.018 *).

- PQ1 “How natural did the interaction with the environment seem?” (*p* = 0.003, see Figure 8)

The results demonstrated that the dynamic condition is significantly different compared to empty (pbonf = 0.004 **) and static conditions (pbonf = 0.018 *), while there is no significant difference between the static and empty conditions (pbonf = 1.000).

**Figure 9:**
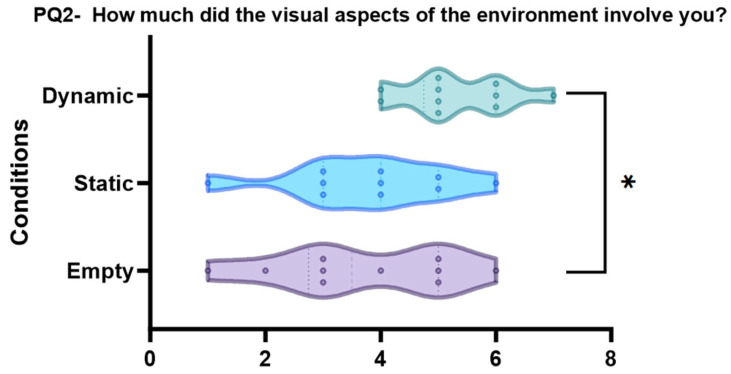
Violin plots and post-hoc comparisons for “How much did the visual aspects of the environment involve you?”. Dynamic scene is significantly better than empty scene pbonf = (0.036 *).

- PQ2 “How much did the visual aspects of the environment involve you?” (*p* = 0.020, see Figure 9)

The dynamic condition is significantly different from the empty condition (pbonf = 0.036 *), while the empty and static conditions are not significantly different (pbonf = 1.000) from each other. Additionally, the dynamic and static conditions are marginally significant (0.054).

**Figure 10:**
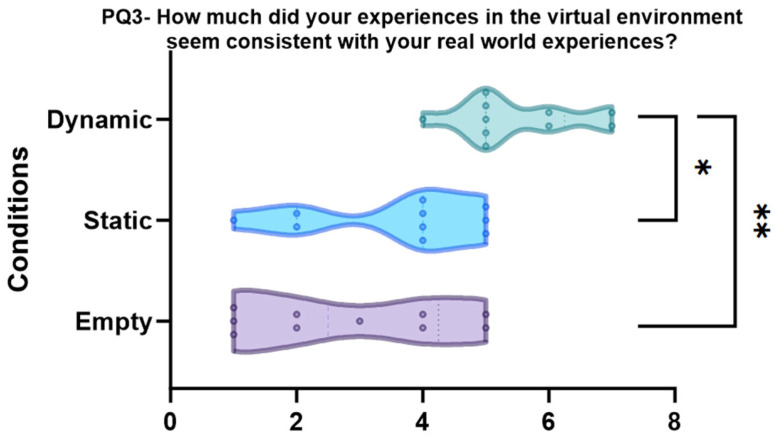
Violin plots and post-hoc comparisons for “How much did your experiences in the virtual environment seem consistent with your real-world experiences?”. Dynamic scene is better than static (pbonf = 0.013 *) and empty (pbonf = < 0.001 **) conditions.

- PQ3 “How much did your experiences in the virtual environment seem consistent with your real-world experiences?” (*p* < 0.001, see Figure 10)

The results indicated that both dynamic (pbonf < 0.001) and static (pbonf = 0.013 *) conditions are significantly different from the empty condition, while there is no significant difference between the empty and static conditions (pbonf = 0.606).

**Figure 11:**
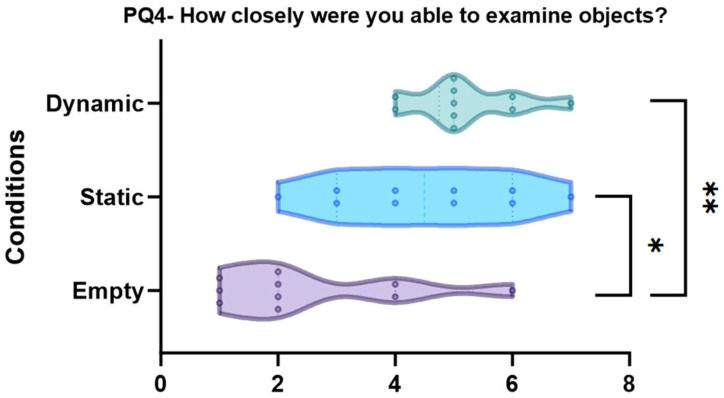
Violin plots and post-hoc comparisons for “How closely were you able to examine objects?”. Dynamic (pbonf < 0.001 **) and static (pbonf = 0.012 *) scenes are better than empty scene.

- PQ4 “How closely were you able to examine objects?” (*p* < 0.001, see Figure 11)

The dynamic condition is significantly different from the empty (pbonf < 0.001 **) condition, whereas there is no significant difference between the dynamic and static (pbonf = 0.842) conditions. Additionally, the empty condition is significantly different from the static (pbonf = 0.012 *) condition.

The other six questions’ answers showed that there is no significant difference between the conditions (the pbonf value is always greater than 0.050). These questions are listed below:

- PQ5 “How completely were you able to actively survey or search the environment using vision?” (*p* = 0.221).
- PQ 6 “How involved were you in the virtual environment experience?” (*p* = 0.056).
- PQ 7 “How quickly did you adjust to the virtual environment experience?” (*p* = 0.446).
- PQ 8 “How much did the visual display quality interfere or distract you from relaxing?” (*p* = 0.975).
- PQ 9 “How much did the auditory aspects of the environment involve you?” (*p* = 0.726).
- PQ 10 “How well could you identify sounds?” (*p* = 0.266).

## 5. Discussion

The results supported only H1, that the dynamic VR CRs increase user presence (H1), and that the experience was perceived as more natural by the user.

Meanwhile, the descriptive analyses of our results demonstrated that the dynamic condition is superior compared to both empty and static conditions in reducing self-perceived stress (−36%), however, the ANOVA results showed that the data were not significantly different (*p* > 0.050).

**Figure 12:**
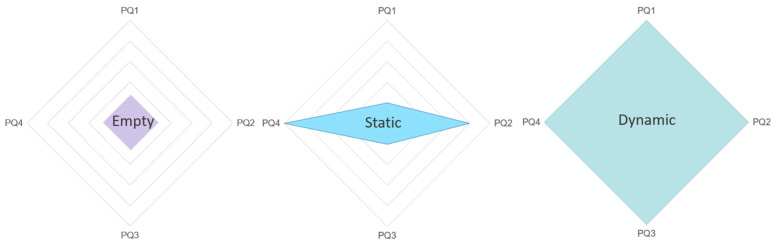
Overview of the Conditions Presence Questionnaire results demonstrated in the radar plot of our three conditions.

We created a radar plot graph to graphically represent our user study outcomes. Considering the significance of the PQ questions we, gave a point to the condition from 1 (lower score) to −5 (better score), see Figure 12.

The results indicate that dynamic scene elements have a positive impact on the user’s presence (PQ1–4). Participants declared feeling more involved and engaged in the dynamic VR condition than in the other conditions. This infers that the traditional chromotherapy rooms may be replaced by dynamic VR experiences in places such as clinics and schools to increase user presence and eventually reduce costs of maintenance and/or space.

User-centric design is essential to emphasize the user’s needs and constraints. Highlighting that different scenarios, such as clinical education and VRCRs, can require diverse design perspectives and specific settings regarding privacy, consent, and the potential unexpected consequences. Therefore, cross-disciplinary collaboration is required, such as among psychologists, designers, and developers, for a holistic approach to the user experience and therapeutic advantages.

VRCRs can become a key component of employee well-being strategy plans by providing an escape from stressful situations, improving overall job satisfaction, and reducing burnout.

We also report below the limitations of the study conducted. One of the main limitations of the study concerns the limited sample size considered, which affects the robustness of the results. However, our study was designed as a preliminary evaluation of the VRCR design elements, and we believe that the results provide valuable insights for future research with larger sample sizes. Indeed, we understand the importance of having a larger sample to confirm the robustness of our results.

Another limitation concerns the way we designed the three conditions, from a visual and auditory perspective. We therefore consider that our results are influenced by the mode in which the three different conditions were designed from an interaction design perspective (i.e., empty, dynamic, and static). In fact, the design elements may affect the user’s perception in relation to the different metrics. We therefore consider, in our future studies, designing the conditions by considering more configurations of visual and auditory stimuli, to deeply evaluate the differences between the conditions.

Another limitation concerns the failure to use instruments capable of measuring users’ physiological signals (e.g., EEG). However, this study represents a preliminary study, and we are currently conducting another user study that presents the same experimental protocol together with the measurement of physiological signals through Muse 2, which are especially crucial for measuring self-perceived stress.

While this work primarily focuses on the influence of VR content on user presence and stress reduction, it is vital to highlight broader implications for humans, especially in stressful daily life struggles such as working life.

Dynamic animated scenes combined with chromotherapy rooms could leverage customized stress reduction therapies. Understanding how dynamic elements affect individuals in coping with stress requires further exploration.

Beyond coping with stress, dynamic VRCRs might stimulate cognitive tasks. Future studies could investigate the long-term effects of dynamic VRCRs on workplace task performance.

User preferences for VRCRs might differ based on the age of the users. Older users may have preferences or be sensitive to certain stimuli.

## 6. Conclusions

In this study, we designed and implemented three VRCRs relaxing scenes using Unity 3D: an empty room (sea and a sky), static (e.g., flowers and ocean texture), dynamic animated (e.g., particle systems, the sea is animated, and adding fractals). All conditions include a musical background, consisting of relaxing low-beta and ocean sounds.

We performed a between-subject (*n* = 30) user experiment to compare the effectiveness of these three different VR CR design conditions on the self-perceived stress and sense of presence of subjects. This experimental design aspect is a novel contribution by comparing VRCR designs and their influence on the user. This comparison is a significant aspect of understanding how design elements impact the user experience of presence and stress reduction. The results supported the idea that the dynamic elements improve user involvement and engagement in the VRCR, enhancing their sense of presence and immersion.

We are encouraged to further explore the dynamic elements of VRCR experiences with a larger number of participants to gain better insight into their psychological state and assess their perceived stress response.

We consider improving future works to be carried out to be eventually supported by the physiological data of the participants’ electroencephalogram (eEG) or heart rate variability (HRV) signals, according to the state of the art.

Then, we want to implement our work on the long-term analysis of VRCRs on users after their use to determine the benefits to users.

## Data Availability

Data are contained within the article.

## Supplementary Materials

The following supporting information can be downloaded at: https://www.mdpi.com/article/10.3390/s24061732/s1, Video S1: Exploiting Virtual Reality for Designing Chromotherapy Rooms. Also available online at https://www.youtube.com/watch?v=QhNW6N3eceA&feature=youtu.be (accessed on 19 January 2024).
